# Tacsac: A Wearable Haptic Device with Capacitive Touch-Sensing Capability for Tactile Display

**DOI:** 10.3390/s20174780

**Published:** 2020-08-24

**Authors:** Oliver Ozioko, William Navaraj, Marion Hersh, Ravinder Dahiya

**Affiliations:** 1 Bendable Electronics and Sensing Technologies (BEST) Group, University of Glasgow, Glasgow G12 8QQ, UK; Oliver.Ozioko@glasgow.ac.uk; 2 Department of Engineering, Nottingham Trent University, Clifton Campus, Nottingham NG11 8NS, UK; william.navaraj@ntu.ac.uk; 3 Biomedical Engineering, University of Glasgow, Glasgow G12 8LP, UK; Marion.Hersh@glasgow.ac.uk

**Keywords:** actuator, tactile sensor, deafblind communication, tactile display

## Abstract

This paper presents a dual-function wearable device (Tacsac) with capacitive tactile sensing and integrated tactile feedback capability to enable communication among deafblind people. Tacsac has a skin contactor which enhances localized vibrotactile stimulation of the skin as a means of feedback to the user. It comprises two main modules—the touch-sensing module and the vibrotactile module; both stacked and integrated as a single device. The vibrotactile module is an electromagnetic actuator that employs a flexible coil and a permanent magnet assembled in soft poly (dimethylsiloxane) (PDMS), while the touch-sensing module is a planar capacitive metal-insulator-metal (MIM) structure. The flexible coil was fabricated on a 50 µm polyimide (PI) sheet using Lithographie Galvanoformung Abformung (LIGA) micromoulding technique. The Tacsac device has been tested for independent sensing and actuation as well as dual sensing-actuation mode. The measured vibration profiles of the actuator showed a synchronous response to external stimulus for a wide range of frequencies (10 Hz to 200 Hz) within the perceivable tactile frequency thresholds of the human hand. The resonance vibration frequency of the actuator is in the range of 60–70 Hz with an observed maximum off-plane displacement of 0.377 mm at coil current of 180 mA. The capacitive touch-sensitive layer was able to respond to touch with minimal noise both when actuator vibration is ON and OFF. A mobile application was also developed to demonstrate the application of Tacsac for communication between deafblind person wearing the device and a mobile phone user who is not deafblind. This advances existing tactile displays by providing efficient two-way communication through the use of a single device for both localized haptic feedback and touch-sensing.

## 1. Introduction

Tactile displays enable people’s interaction with the environment by creating tactile sensation on the skin as haptic feedback which is perceived and interpreted by the brain. Haptics, which involves both tactile (cutaneous) and kinesthetic (force) feedback, plays a great role in the way we communicate, interact with and perceive the environment [1,2,3,4]. In the area of robotics and virtual reality, it’s viewed as real and simulated touch interactions between robots and humans [5]. This means that these interactions may be accomplished by human, machine or both and the environments can be real or virtual. This is recently changing the way humans interact with information and communicate ideas [6,7]. In case of haptic visual aids for deafblind people, the interaction is not accompanied by vision or hearing.

The human sense of touch is well developed and there is often a minimal perceived intensity for various stimuli like pressure, temperature, heat, and vibration. Humans are able to recognize common objects by touch within 1–2 s and the skin is very sensitive to light pressure [8]. Although there are different mechanoreceptors, tactile reception results from a combination of all the receptors in a particular skin area. The threshold of tactile perception depends on several factors like location, contact area, type of stimulus, duration, age and even hormone levels [9,10,11,12,13,14]. In [12], it was observed that the most significant age difference in vibrotactile detection threshold was found in the glabrous finger with the difference being less pronounced at lower frequencies (5–10 Hz) and more pronounced as the frequency increases to 300 Hz. Studies have equally shown that the perceived intensity of stimulation is determined by both the depth of penetration and the rate of skin indentation [8,15]. The large numbers of touch receptors present in our skin allow us to obtain rich information through haptic interaction by touching the objects around [16,17,18,19,20,21]. So, the importance of touch sensing [22] is evident in situations where other sensing modalities such as vision are insufficient or unavailable [23]. An example is the interaction by the hearing and visually impaired people in the real-world environment [24,25,26,27]. Through various forms of tactile stimulations such as skin stretching [28,29], vibration, force or painless electric shock, they substitute the hearing and vision impairments. Thus, they substantially rely on the tactile sensing [30] as well as tactile feedback (e.g., using actuators in tactile displays) to explore and manipulate objects around them [6,7,31]. Similarly, combination of tactile sensing [32] and tactile feedback is needed in areas such as minimal invasive surgery [2,5] and virtual reality [33,34] to improve the user’s haptic interaction experience. Therefore, a tactile display with an inherent ability to provide touch feeling as well as the vibrotactile feedback within these limits would be advantageous. Further, considering the close contact of such displays with the curvy human body, they are desired to be soft and flexible [35,36].

Several assistive tactile displays with a single actuator or array of actuators have been developed for the purpose of providing tactile feedback. Early research in this area mainly focused on sensory substitution for the blind using Braille as well as camera-based types such as opticon [37] which reproduces texts in tactile form. However, the application domain has recently expanded to fields such as robotics, [38] haptics, teleoperation, virtual reality, video games, and prosthetics etc. Although all tactile feedback presents certain information to the user, the difference between tactile information for sensory substitution and information for robotic surgery is obvious. Here, we have grouped these existing tactile displays as: (a) the exploration types (ET), in which the user explores the surface of objects as in Braille, and (b) the localized stimulation types (LST), in which the actuator is positioned on the palm or elsewhere on the body to exert localized stimulation such as vibration, or skin stretch. The latter is popularly used for assistive purposes [27,39,40].

Table 1 summarizes different types of tactile displays and the actuation technologies explored so far. These include electromagnetic, electrostatic, dielectric elastomer (DE), piezoelectric [41], pneumatic, rheological fluids, and shape-memory alloy (SMA) [41]. Each of these technologies has advantages and disadvantages with regards to the development of tactile displays. Electrostatic actuators which are based on the attraction of two forces are widely used due to their low power requirements and fast actuation speed but suffer limited spatial resolution and performs better on dry fingers. Dielectric elastomers are excellent smart materials for actuation and are widely explored for a number of applications including soft robotics and tactile displays for assistive purposes. However, the common drawback of DE actuators is the requirement of very high voltage (usually in the order of kV). SMA provides high force and large displacement but has characteristic slow response time and suffers hysteric effect. Another technology that provides high force and large displacement is the pneumatic-based tactile displays, but they are often quite bulky and the lack of portability poses a problem for wearability. In comparison with these technologies, the electromagnetic principle used here to develop the integrated sensor and actuator offers high precision and displacement at comparatively low voltages (Table 1), which are important for wearable applications. Additionally, none of the tactile displays (e.g., Braille displays and deafblind smart gloves) reported so far have the dual integrated capability of tactile sensing and vibrotactile feedback.

**Figure 1:**
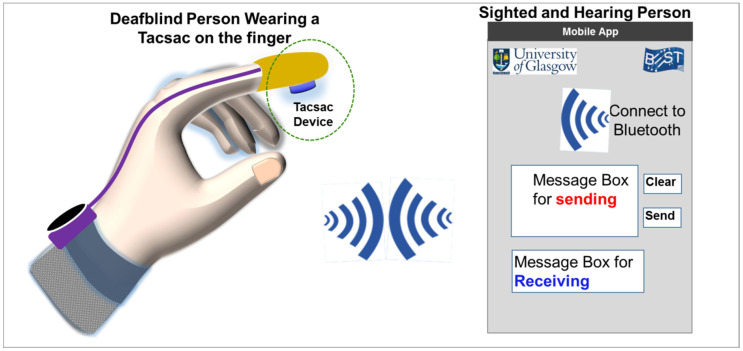
Application of the Tacsac device.

Here we present a device (Tacsac) with both capacitive tactile sensing capability and vibrotactile feedback for application in tactile displays (LST); e.g., assistive communication devices by the deafblind people who otherwise use tactile communication methods such as deafblind manual alphabets [53]. It consists primarily of two modules; (1) a capacitive touch sensing module fabricated using flexible printed circuit board; and (2) an actuation module based on electromagnetic principle which is capable of providing vibrotactile feedback at a range of frequencies (10 Hz–200 Hz) within the range perceivable by human. A mobile app was also developed and utilized for the demonstration of its potential application as a means of communication. In particular, the presented device could be used by deafblind to communicate with people without vision/hearing impairment via a smartphone as we demonstrate here (Figure 1). The capacitive touch sensing layer serves as a touch interface for sending message to the mobile phone user (via the developed mobile app), while the vibrotactile module is used as a means of interpreting the message to the user.

Further, the Tacsac device is advantageous as it utilizes a skin contactor which directly stimulates the skin of the user. This enables the provision of localized vibration rather than full-body vibration like popular conventional commercial vibration motors used so far in deafblind communication devices.

## 2. Materials and Methods

### 2.1. Device Structure and Operating Principle 

**Figure 2:**
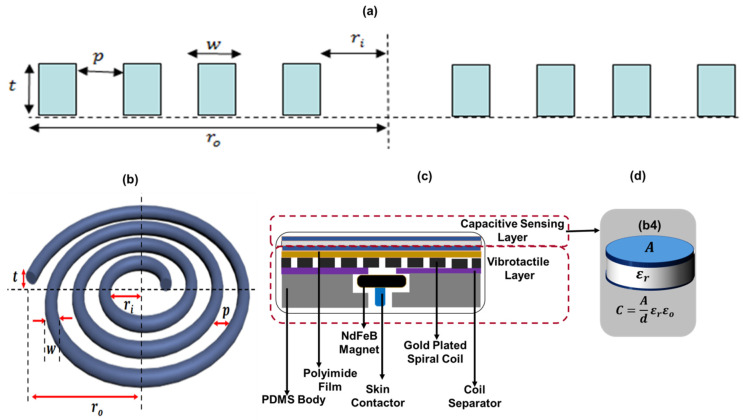
The structure and working principle of Tacsac (**a**) 2D axisymmetric structure of the spiral coil (**b**) Structure of the spiral coil (**c**) Structure of the Tacsac device. (**d**) Capacitive Sensing Layer.

Figure 2 shows the structure and working principle of the Tacsac device. It comprises two main modules: (a) the touch-sensing module, and (b) the vibrotactile module. The touch sensing module was designed with a simple metal-insulator-metal capacitive configuration with capacitance expressed as:

(1) $$C=\varepsilon_{r}\varepsilon_{0}A/d$$

where *C* is the capacitance, which changes when the sensor is touched, *ε_r_* is the relative permittivity of the dielectric material, *ε_0_* is the permittivity of free space, *A* is the area of metal plates and *d* is the distance between the two conducting metal plates. The actuation module uses electromagnetic principle and is driven by the interaction of a spiral coil and a permanent magnet. As a principal component of the actuator, the coil determines the magnetic field produced. When a uniform square current pulse flows through the coil at a particular frequency, a pulsating magnetic field is produced along the axis, which exerts a force on the tiny magnet of the actuator and leading to periodic actuation of the top layer. The choice of the parameters of the coil is very key in the realization of a spiral coil which is capable of giving the desired results. Here we present how different parameters of the coil were calculated.

Any wire carrying current creates a magnetic field in the region surrounding it and the relationship between the current and the accompanying magnetic field is given by Biot Savart’s Law. If a spiral coil carrying current *I* is considered to be made up of *n* number of concentric circular loops with inner radius *r_i_*, outer radius *r_o_* and thickness *t* (Figure 2a,b), then if *n = N* where *N* = number of turns of the spiral.

Utilizing the 2D-axisymmetric model of the spiral coil as shown in Figure 2a (adapted from [54]), the relationship between the internal and external radii of the spiral coil is given as:

(2) $$r_{o}-r_{i}=N\left(p+w\right)$$

where p is the pitch of the coil, w is the width of the coil conductor. The coil aspect ratio is also an important consideration in as different coil aspect ratio could affect the magnetic field produced at the center of the coil which provides the required force of actuation when it interacts with a magnet. Defining α=w/t as the coil aspect ratio we have:

(3) $$r_{o}-r_{i}=N\left(p+\alpha t\right)$$

Given the length of the spiral coil as $l=N2\pi r_{o}$, we derived the total magnetic field generated at the center by the *N*-turns of the spiral as:

(4) $$B_{Center\;of\;Spiral}=\overset{N}{\underset{1}{\sum }}\frac{\mu_{0}I}{2\left(N(\alpha t+p)\right)}\ln\left(\frac{r_{o}}{r_{o}-N\left(\alpha t+p\right)}\right)$$

where, $\mu_{0}$ is the relative permeability of free space (4*π* × 10^−7^) *[H/m]*. Equation (4) depicts the relationship between the coil parameters and shows how varying these parameters affect the magnetic field generated at the center of the actuating coil.

### 2.2. Device Fabrication

This section presents the fabrication of the Tacsac device, which involves three main tasks: (1) fabrication of the spiral coil, which is the primary element of the integrated actuator that provides the vibrotactile feedback; (2) fabrication of the capacitive sensing layer; and (3) Integration of the vibrotactile actuator and the capacitive sensing layer to realize Tacsac.

#### 2.2.1. Fabrication of the Spiral Coil

**Figure 3:**
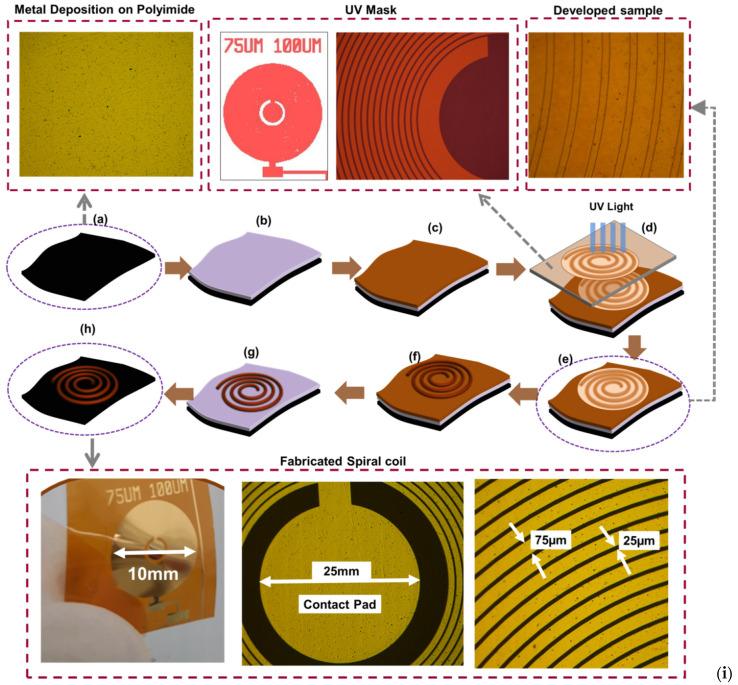
Fabrication steps for realization of the coil: (**a**) Initial flexible substrate; (**b**) Gold deposition; (**c**) Spin-coating of photoresist; (**d**) Exposure of photoresist; (**e**) Developing the photoresist; (**f**) Electroplating the coil; (**g**) lift-off the photoresist; (**h**) Etching of the seed layer; and (**i**) Fabricated Coil.

The spiral coil is the primary element of the actuating layer and was fabricated using the Lithographie Galvanoformung Abformung (LIGA, Figure 3) process [55], which is needed to produce structures with high aspect ratio. A Plassys MEB 550S Electron Beam Evaporator system (Plassys, Glasgow, UK) was used to deposit a 20 nm/80 nm NiCr/Au on a flexible 50 µm polyimide sheet. Following this was the spinning of an AZ4562 photoresist (Clariant GmbH, Wiesbaden, Germany) at 2000 rpm for ~3 s, and the sample left at room temperature for ~30 min prior to baking on a hot plate at ~100 °C for 10 min. The baked sample was left again at room temperature for 30 min to allow evaporation of the solvent. This was followed by an exposure of the sample to ultraviolet (UV) light for ~60 min following a standard lithography technique. An AZ826 developer was utilized to develop the exposed sample for ~10 min, after which it was rinsed in reverse osmosis water.

In order to increase the thickness of the resulting spiral coil, we electroplated it using a litre of non-cyanide gold electroplating solution. The solution was prepared by first heating 250 mL of RO water at 50 °C using a hotplate. The water was then removed from the hotplate and 60 mL of solution containing Na_2_SO_3_ and tripotassium citrate (K_3_C_6_H_5_O_7_) was added to the water. Following this, 5 mL of brightener (containing arsenic salt) was added. Next is the addition of 50 mL of gold complex solution which contains gold sulphite. Finally, the resultant solution was adjusted to reach 1liter by adding more RO water.

The coil sample was then electroplated for ~45 min using the prepared non-cyanide gold complex solution [56] to realize a coil with ~17 µm thickness. The unwanted gold layer was then etched using conventional gold etchant for ~15 s, thus exposing only the NiCr seed layer. The sample was annealed at 350 °C in a furnace for ~20 min under a nitrogen atmosphere to increase the strength of electroplated metal and avoid undesirable lift-off during etching of the seed layer. The NiCr seed layer was then was removed using Nichrome etchant to realize the required spiral coil as shown in Figure 3h.

#### 2.2.2. Fabrication of the Capacitive Sensing Layer

**Figure 4:**
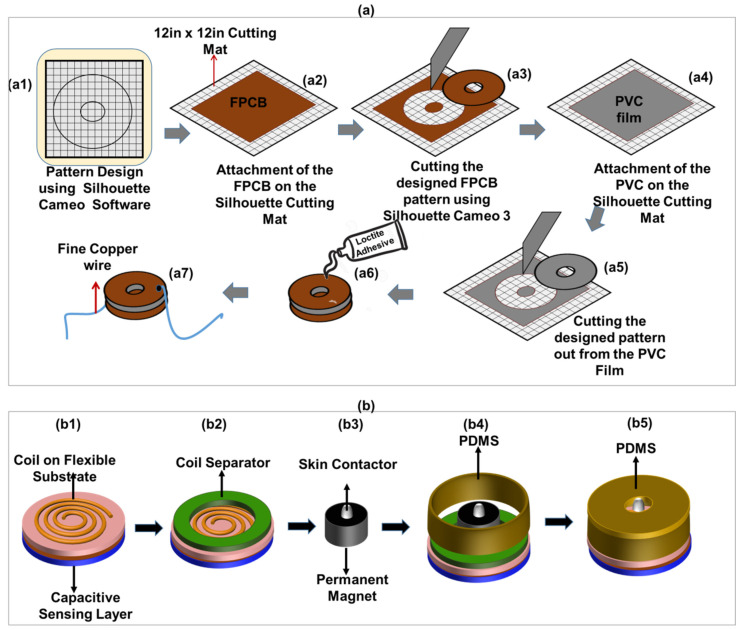
(**a**) Fabrication steps for the sensing layer; (**a1**) Design of the pattern using CAD software; (**a2**) attachment of the FPCB on the 12 in × 12 in cutting mat; (**a3**) Cutting of two layers of the pattern using the Silhouette Cameo 3 blade cutter; (**a4**) Attachment of the PVC film on the 12 in × 12 in cutting mat (**a5**) Cutting of the PVC film using the Silhouette Cameo 3 blade cutter; (**a6**) Bonding of the FPCB and PVC layer using Loctite adhesive; (**a7**) Soldering of a fine copper wire on both layers of the FPCB to serve as electrode; and (**b**) Fabrication steps for integration of the capacitive sensing layer and vibrotactile actuator(**b1**) Attachment of the touch-sensing layer to the coil; (**b2**) Integration of the coil separator; (**b3**) Attachment of skin contactor to the permanent magnet; (**b4**) Integration of the PDMS packaging; (**b5**) Final packaging of the actuator using PDMS cover.

The touch-sensing module was realized following the steps depicted in Figure 4a. It was fabricated using a facile planar capacitive structure realized by bonding two layers of single-sided flexible printed circuit board (FPCB) with ~35 µm of copper on a polyimide substrate. This was achieved by sandwiching a PVC film between the non-conducting surface of one FPCB with the conducting copper surface of the other, and so the polyimide substrate of the top FPCB and the PVC serves as the dielectric layer for the capacitive touch sensor (Figure 4a). The FPCB and PVC were cut using the Silhouette Cameo 2 blade cutter (Silhouette, Lindon, UT, USA, Figure 4(a3,a5)). The software of the Silhouette Cameo has a built-in library of different materials with options of editing their properties. The pattern to be cut was first designed in a graphic software and then transferred to the Silhouette Cameo software for cutting Figure 4(a1). The Silhouette Cameo was set to be able to cut only the required portions of the sheet. To do this, the speed, force, and blade position of the blade cutter was set to 5, 20, and 10 cm·s^−1^ respectively. This was followed by attachment of a plain FPCB on a sticky 12 in × 12 in cutting mat, from which the designed pattern was cut out Figure 4(a3). Similar steps were followed to cut a PVC film (Figure 4(a4,a5)). After cutting, the unwanted parts were removed revealing only the pattern, as shown in Figure 4(a5). The cut piece of PVC was sandwiched between two cut pieces of FPCB as shown in Figure 4(a6). This was then followed by the attachment of a fine copper wire to serve as electrodes Figure 4(a7).

#### 2.2.3. Device Integration

The steps for the realization of the touch-sensitive actuator (Tacsac) are shown in Figure 4b. The main components of the actuator include the touch sensitive layer, the coil, a permanent magnet, skin contactor, and PDMS packaging. The integration and packaging was carefully designed to reduce damping of the actuation/vibration. The actuator has an overall diameter of ~1.5 cm and was designed to fit appropriately into the user’s finger. It was realized layer by layer as shown in Figure 4b. A cylindrical mould with 1.5 cm and 1.9 cm inner and outer diameter respectively was designed and realized with 3D printer for the PDMS packaging. The mould was designed for the body of the actuator and meant to realize a PDMS packaging of diameter 1.5 cm and height 0.4 cm, with a hole of 1mm inner diameter for the movement of the skin contactor.

For the realization of the actuator, 10:1 PDMS (Sylgard 184, Sigma-Aldrich, Gillingham, UK) comprising of mixture of pre-polymer base and crosslinking agent was prepared and poured into the mould, and then cured at 80 °C in the oven for 15 min. The same PDMS was also used to attach the touch-sensitive layer to the coil and cured for 10 min Figure 4(b1). The coil separator was then attached to the coil substrate Figure 4(b2) using Loctite transparent adhesive (Amazon UK, Slough, UK).

The permanent magnet used here was a 2 mm thick N42 grade neodymium magnet from E-Magnets (E-Magnets, Berkhamsted, UK), comprising of ~29–32% neodymium, 64.2–68.5% iron and 1–1.2% E-Boron (NdFeB). A 1 mm^2^ skin contactor (made of polylactic acid (PLA) plastic) was attached to the permanent magnet using a Loctite transparent adhesive, and both placed on the coil separator (Figure 4(b3)). The actuator was then packaged with the cured PDMS described earlier in this section (Figure 4(a4,a5)).

### 2.3. Device Characterization

**Figure 5:**
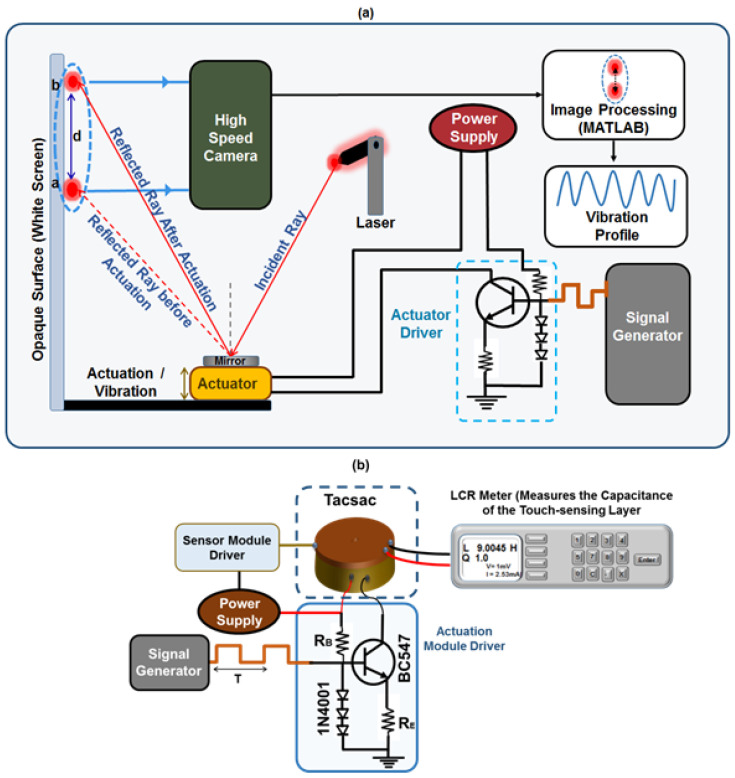
(**a**) Setup for Characterization of the Actuation Module (**b**) Setup for the characterization of sensing module of the fabricated Tacsac with and without actuation.

This section describes the procedure for characterizing the actuation (vibrotactile) and sensing (capacitive) capabilities of the Tacsac device. The actuation characterization (displacement and amplitude characteristics at different frequencies) of the actuator in response to varying current pulse inputs was carried out by employing custom-made optical lever technique (Figure 5).

In this technique, a pointed laser ray is directed onto a reflective material (mirror) placed on the actuator and the reflected ray properly directed on to an opaque screen where the actuation is measured as a magnified value of the original displacement (Figure 5). Before the experiment, the laser and the actuator were all firmly attached to a stable optical table and the ray adjusted to obtain a sharp image on the screen. The actuator was then connected to a power supply, and a signal generator via a custom-made constant current source (Figure 5a). During the experiment, the actuator was driven with different currents (60, 90, 120, 150, and 180 mA) and at different frequencies ranging from 10 Hz to 200 Hz in order to understand the behavior of the actuator over this range which is detectable by human. To record displacement at any time, the camera is used to capture the motion of the ray on the screen when the actuator is turn on at the given current and frequency. In our experiment, the motion of the reflected laser spot on the opaque screen during the vibration of the actuator was recorded as a video using a high-speed camera with 960 frames per second (fps). This enabled the amplitude and displacement characteristics of Tacsac to be effectively captured in real time for different vibration frequencies ranging from 10 Hz to 200 Hz. Further, the recorded videos were processed (with appropriate calibration) using digital signal processing by a MATLAB program to obtain the dynamic response of the Tacsac device.

The capacitive touch-sensing layer was equally characterized to understand how the device responds when touched and when not touched. Force sensing was not studied here since this is not key for the intended application. This characterization was carried out using an E4980AL precision LCR meter (Keysight, Santa Rosa, CA, USA) and a LabVIEW 2018 Robotics v18.0f2 (National Instruments, Austin, TX, USA) program installed in a computer for automatic reading of the capacitance values (Figure 5). Capacitive values were read with the LabVIEW program for the case when actuator was touched and the vibration OFF and then ON. Similar setup was used to drive the actuator in Figure 5 at 150 Hz using a square wave of 5 Vp-p, 50% duty cycle and zero offset (Figure 5). While the actuator was vibrating, the capacitive sensing layer was touched at different intervals using a finger and the change in capacitance was recorded using the LCR meter via the LabVIEW program.

## 3. Results and Discussion

**Figure 6:**
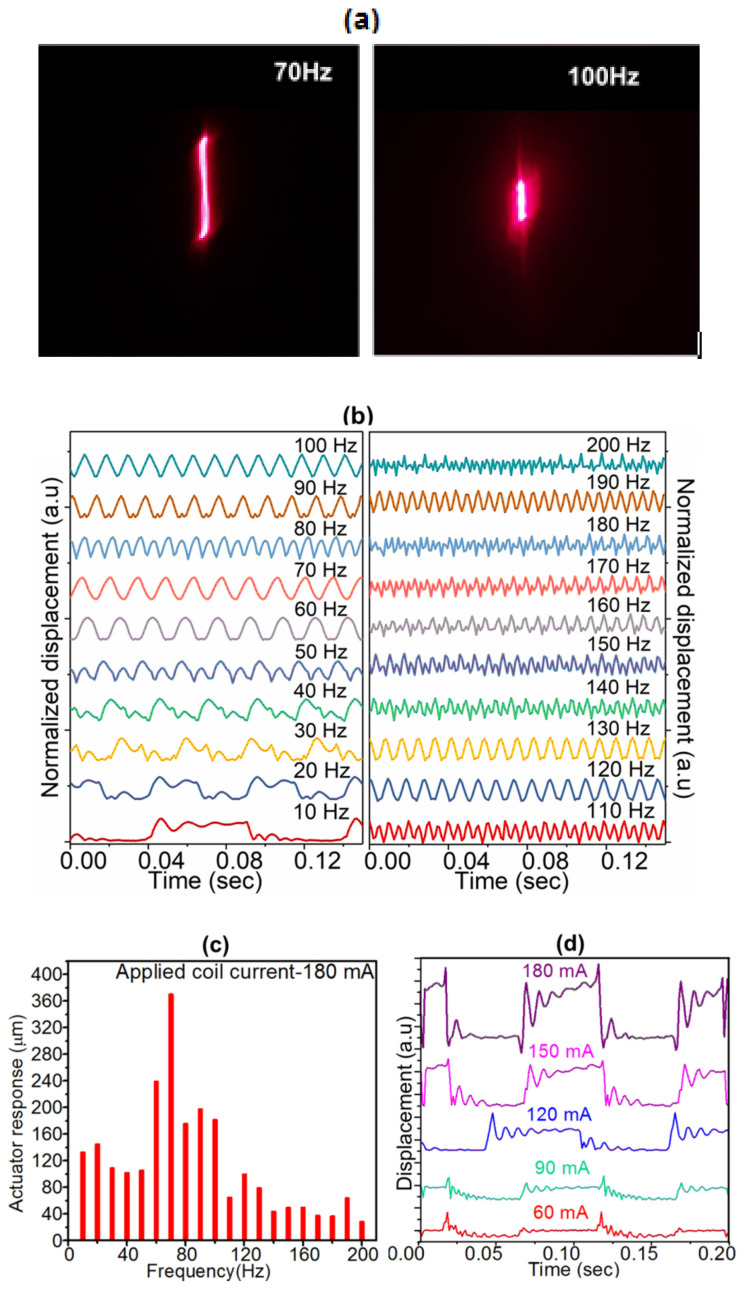
(**a**) Recorded amplitude of the actuator oscillation at 70Hz and 100Hz (**b**) Normalized transient displacement response of the actuator and (**c**) Peak actuator displacement as a function of applied frequency at a current of 180 mA (**d**) Transient response of the actuator at different currents and input frequency of 10 Hz.

Figure 6a shows the captured laser images for recorded amplitude of the actuator oscillation at 70 Hz and 100 Hz. Similar images captured for other frequencies were processed in MATLAB to determine the actual dynamic response of the actuator. Figure 6b shows the normalized transient response profiles of the actuator as a function of elapsed time for a peak pulse current of 180 mA. The actuator displacement was measured for various frequencies ranging from 10 Hz to 200 Hz with equal intervals of 10 Hz which is well within the range detected by human. At low frequencies it can be observed that, as the magnet switches from one position to another, it undergoes damped oscillations before coming to stabilize in that particular position. For frequencies above 50 Hz, the magnet switches smoothly from its position before coming to a steady state. This results in the actuator not getting sufficient time to restore to its equilibrium state before the subsequent input current pulse. The switching pulse may result in constructive or destructive interference with the damped oscillations, resulting in an oscillatory behavior.

Figure 6c shows the absolute amplitude (displacement) of the actuator for different frequencies (within the range detectable by human) at constant current of 180 mA, with the maximum displacement observed at 70 Hz. As the frequency increases further, the displacement drops significantly owing to the inherent mechanical impedance during oscillations. The resonance frequency of the oscillator is in the range of 60–70 Hz. The slight increase of actuator displacement at 120 Hz and 190 Hz is likely related to the first and second overtones of the resonance frequency between 120–130 Hz and 180–190 Hz respectively. Our control experiments at different currents and fixed frequency showed that the actuator response is similar, except proportional increase in amplitude (Figure 6d). During this experiment, the current in the spiral coil was increased to observe its effect on the actuation and to choose the optimum coil current for perceivable vibration. This is because, the magnetic field generated along the axis of the coil increases with pulsed current passing through the coil, leading to stronger repulsive impulse force on the magnet to displace it from its mean position. The maximum input current to the coil was restricted to 180 mA owing to the dimensional limit of the casing of the actuator on the amplitude of actuation. Increasing the current to very large values (>200 mA) could increase the chances of the coil getting hot as result of Joule heating. At a constant current of 180 mA, the actuator was able to give an off-plane displacement of 0.377 mm with negligible Joule heating effect. The transient response of the actuator was equally recorded for various coil currents (60, 90, 120, 150 and 180 mA) at a fixed frequency of 10 Hz (Figure 6d). This showed similar oscillating pattern in each case with current and displacement having direct proportionality. This means that 60 mA gave the least peak to peak displacement while 180 mA gave the highest in this case (Figure 6d). The actuator has a response time ~93 ms (at 180 mA, for as frequency as low as 10 Hz) which is similar to that of conventional vibration motors (~50–140 ms).

**Figure 7:**
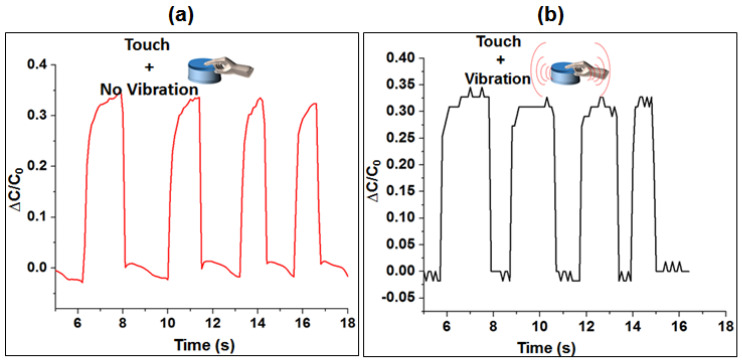
Response of the capacitive sensing layer with and without actuation; (**a**) Sensor output when touched and vibration is OFF; (**b**) Sensor’s output when touched and vibration is ON.

The response of the sensor module characterization, recorded using an LCR meter and a LabVIEW program, is shown in Figure 7a,b. Capacitance values were read when the actuator vibration was OFF and ON. The result shows average ∆C/Co ~ 0.35 for both cases. This means that the sensing layer is able to respond quite similar both when the actuator is ON or OFF. The results were similar other frequencies tested and so only the response of the actuator at frequency of 150 Hz is presented as shown in Figure 7.

## 4. Application

**Figure 8:**
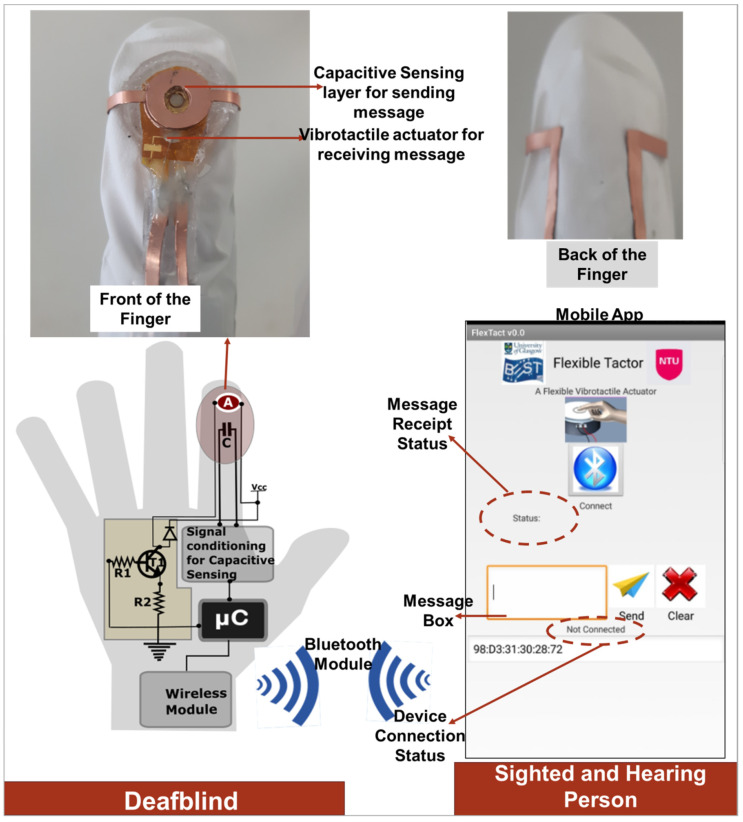
Application of Tacsac for wireless communication of deafblind people with mobile phone users.

Figure 8 shows the block diagram and result of the actuator with the mobile app developed in this work. This is to demonstrate one of the applications of Tacsac, which is wireless communication between (1) a deafblind person and a hearing-and-sighted person who uses a mobile phone, or (2) deafblind-to-deafblind people, both of whom wear the device. The overall communication system comprises four main modules (1) the fabricated Tacsac device, (2) the control module including the drive and readout circuits, (3) the wireless module, and (4) the developed mobile app.

The deafblind user employs the actuator to communicate with a mobile phone held by the sighted and hearing person. Messages are sent by the deafblind person using the capacitive sensing layer of the actuator, and then received via the vibrotactile actuator in the form of vibration. This could also be adapted for use as a Morse code communication device for deafblind people. Morse code is one of the methods used by deafblind people for communication and this device could be of benefit for users of this communication method. In this case, messages sent from the mobile app would be converted to Morse codes in the form of vibration of varying frequencies and duration. To send messages in the form of Morse code, deafblind people could tap the capacitive sensing layer, which would be decoded and converted to text messages by the mobile app. In comparison to Braille for instance, Morse code has advantage in terms of wearability and simplicity and with only a single device (integrated sensor and actuator), a two-way communication can be established. Braille introduces some level of complexity in terms of the number of devices required to represent the six dots of Braille.

However, in this work we have only demonstrated the use of Tacsac to successfully send and receive message from the developed mobile app with a single fabricated actuator. This demonstration was achieved using a mobile app which communicates with the actuator via an HC-05 Bluetooth module (Amazon UK, Slough, UK). When the app is launched, the user presses the connect button (Figure 8) to connect with the actuator and a connection status is displayed (Figure 8). To communicate from the app to the Tacsac device, the user types a number in the message box of the app and sends it via Bluetooth. When the actuator receives this information, it vibrates accordingly. For instance, when the number “1” is sent from the app, the actuator vibrates once, while it vibrates twice when the number “2” is sent. To demonstrate the communication from Tacsac to the mobile app, the user touches the capacitive sensing layer, this touch is then sensed, and the information sent to the mobile app via Bluetooth (Supplementary Materials Video S1). When the mobile app receives this information, it displays the word “Touched” or “None” otherwise (see message receipt status in Figure 8).

## 5. Conclusions

In this work, an assistive haptic device for application in tactile displays (such as smart assistive gloves) is presented. The actuation is based on electromagnetic principle and the device also has capacitive touch-sensing capability. Considering the state of the art, this is a step forward given that majority of the existing tactile displays do not have the ability for both touch sensing and vibrotactile feedback. The addition of touch-sensitivity to the tactile displays is advantageous for simultaneous sending and receiving of information―particularly for deafblind people as it will provide a close-loop communication system. The sensor and actuator combination show good performance and the potential to be used in a tactile display for deafblind communication. The integrated actuator provides a 0.377 mm displacement and its measured vibration profiles showed its capability to provide perceivable vibration within a wide range of perceivable frequencies (10 Hz to 200 Hz). A developed app was used to send and receive messages to/from the actuator, demonstrating its application for communication between deafblind people and mobile phone users who have hearing and vision. This work finds application in Morse code communication for deafblind people where the user is able to compose messages based on Morse code and then send it to mobile phone user. Future work will involve the fabrication of array of such integrated sensor and actuator for application in other methods of deafblind communication (e.g., British deafblind manual alphabet).

## Figures and Tables

**Table 1:** Summary of selected tactile displays and their actuation technology.

Technology	Tactile Sensing	Disp. (µm)	Type	Current Req. (mA)	Voltage Req.	Freq (Hz)	Ref.
Piezoelectric	No	0.257	ET	<250	80 Vpk	250	[41]
Electromagnetic		1000	ET	0.250	5 V	N/A	[42,43]
Electrostatic	No	N/A	ET	<200	2000 Vp–p	100–400	[44]
Electroactive Polymer	No	680	LST	N/A	5 kV/mm	0.1–300	[45]
Thermal		61	ET	N/A	28.7 V	N/A	[46]
Electrotactile	No	N/A	ET	N/A	9.3–63.4 V	20	[47]
ERM Motor	No	NA	LST	72	5 V	208	[39,48]
SMA	No	up to 2000	ET	N/A	120 mA	2	[49,50]
Pneumatic	No	560	ET	N/A	N/A	0.2–20	[51]
Electrorheological Fluid	No	700	LST	N/A	4 kV	10	[52]
Electromagnetic	Capacitive	377	LST	100 mA	3–5 V	10–200	This Work

N/A―Not Available. ET-Exploration Type; LST―Localized Stimulation Type; ERM―Eccentric Rotation Mass; SMA―Shape Memory Alloy.

## Supplementary Materials

The following are available online at https://www.mdpi.com/1424-8220/20/17/4780/s1, Video S1: Tacsac communicating with a custom-made mobile app.
